# Factors associated with quality of life among elderly patients with type 2 diabetes mellitus: the role of family caregivers

**DOI:** 10.1186/s12889-024-17917-z

**Published:** 2024-02-21

**Authors:** Haijing Zan, Zhixing Meng, Jing Li, Xinjian Zhang, Tao Liu

**Affiliations:** School of Nursing, Jinzhou Medical University, Linghe District Jinzhou City, No.40,Section 3, Songpo Road, Jinzhou, Liaoning Province People’s Republic of China

**Keywords:** Type 2 diabetes mellitus, Elderly, Quality of life, Sense of coherence, Caregiver competence, Family caregiver

## Abstract

**Background:**

As a long-term chronic disease, Type 2 diabetes mellitus (T2DM) patients’ quality of life is affected by both themselves and his/ her close relatives, requiring comprehensive support from family members to ensure that patients are able to manage disease. The objective of this study is to investigate the relationship between caregivers’ sense of coherence, caregiver competence, and T2DM patients’ quality of life, as well as to explore the factors affecting patients with T2DM patients.

**Methods:**

This investigation was a cross-sectional study. Between October 2022 and July 2023, 392 participant-caregiver dyads from two hospitals in Jinzhou City, Liaoning Province, were researched. Participants were investigated by General Characteristics Questionnaire, Modified Barthel Index (MBI), Diabetes Specific Quality of Life Scale (DSQLS), Sense of Coherence scale-13 (SOC-13), and Family Caregiver Task Inventory (FCTI). Data were statistically analyzed using SPSS 25. Univariate and multivariate linear regression analyses were used to identify the independent factors associated with the quality of life of elderly patients with T2DM.

**Results:**

The average score of T2DM quality of life was 61.14 (SD = 7.37), quality of life was negatively correlated with sense of coherence (*r*=-0.344, *P*<0.01) and positively correlated with caregiver competence (*r* = 0.522, *P*<0.01). Furthermore, we found that age, disease duration, activities of daily living scores, sense of coherence, and caregiver competence scores were the main predictors of quality of life (R^2^ = 0.375, *P* < 0.001).

**Conclusions:**

This study found that high levels of sense of coherence and caregiver competence in family caregivers were associated with better quality of life for patients. Furthermore, we also found that good quality of life was also related to younger age, shorter disease duration, and less dependence. This study offers a feasible example for policymakers to improve the quality of life from the perspective of T2DM patients’ family caregivers.

**Supplementary Information:**

The online version contains supplementary material available at 10.1186/s12889-024-17917-z.

## Background

Diabetes is now a major public health concern on a global scale. The International Diabetes Federation (IDF) estimates that 536.6 million people are living with diabetes in 2021, and this number is projected to increase by 46%, reaching 783.2 million by 2045. It was reported that China had 141 million diabetics in 2021, making it the country with the biggest diabetic population worldwide [1]. Type 2 diabetes mellitus (T2DM), one of the most common forms of diabetes, accounts for about 90% of the total, especially in the middle-aged and elderly population [2]. In China, the prevalence of T2DM in the elderly is 30% [3]. With age and complications, patients are often accompanied by a variety of functional impairments and physical limitations ultimately leading to a reduced quality of life [4].

Quality of life (QoL) has received significant attention as a crucial medical result in recent years and it is the measure most frequently used to assess the effects of chronic illness or its treatment. According to the World Health Organization (WHO), QoL is an individual perception of their position in life in the context of the culture and value systems in which they live and about their goals, expectations, standards, and concerns [5]. According to a study by Qi et al., elderly Chinese individuals with type 2 diabetes have a poor quality of life [6]. At present, most researches have been more concerned with how patients can enhance their quality of life on their own and less concerned with the effects of outside variables on patients [7–9]. However, the role of the family caregivers is not to be overlooked either. They are an important extension of the health care system. As Bowen Family System Theory states that the family should be viewed as a system and family members are an important part of the family system, which implies that group members are interconnected and interdependent [10]. The patient is also affected, either more or less, by the feelings and actions of family members. According to traditional Chinese culture and medical resources, the major of patients with chronic illnesses and senior citizens typically receive care at home and in the neighborhood. Because of the complexity of diabetes management, some patients will be more reliant on their caregivers than others [11]. For those with T2DM, this means that the job of the family caregiver is becoming more and more crucial.

Over time, family caregivers may gradually feel stressed and burdened [12]. Protective psychological factors can reduce the negative impact of caregiving. Sense of coherence (SOC) is a positive psychological resource that reflects an individual’s ability to cope positively with stress in the face of life’s pressures and stimuli [13]. The concept emerged from Antonovsky Salutogenic Theory, which outlines the unique way in which each person views the world and their circumstances as comprehensible, manageable, and meaningful [14]. A longitudinal study indicated that SOC can mediate caregiver stress and psychological discomfort [15]. Another study identified that active psychoeducation of family caregivers can be effective in enhancing caregiving [16].

Caregiver competence, which has a direct impact on the standard of at-home care, is the ability of main carers to provide proper daily care, look for disease-related knowledge and skills, and provide mental and emotional support to care receivers [17]. The finding of a study in Northern Thailand demonstrated the influence of diabetes knowledge and behaviors among family caregivers on patients’ QoL [18]. The prognosis and QoL of patients are significantly impacted by the caregiving competence. Enhancing one’s ability to provide care lessens the burden of providing it, which is crucial for lowering hospital admission rates and conserving healthcare resources.

To our knowledge, there are few reports on the relationship between family caregivers’ SOC, caregiver competence and QoL of patients. In addition, current research has focused on caregivers of patients with cancer, stroke, and dementia and less on T2DM caregivers [19, 20]. Therefore, this study considered the inclusion of family caregivers in the investigation of factors affecting the QoL of elderly patients with T2DM and explored the three variables, to provide a reference for improving the QoL of elderly patients with T2DM from the perspective of family caregivers.

## Methods

### Design and procedure

This cross-sectional study was performed from October 2022 to July 2023. According to Kendall’s sample calculation method [21], sample were measured to be 5–10 times the number of variables. The General Information Questionnaire consists of 18 entries, 4 dimensions of the Quality of Life Scale, 3 dimensions of the Sense of Coherence Scale, and 5 dimensions of the Caregiving Competence Scale, for a total of 30 variables, taking into account a 20% sample loss. As a result, the calculated sample size was at least 180–360. A total of 400 questionnaires with missing data were distributed in this study, and 392 valid questionnaires were recovered, with an effective rate of 98%, meeting the sample size requirements. Therefore, 392 participant-caregiver dyads were investigated from two hospitals in Jinzhou City. Patients must meet the following criteria: at least 6 months with physician-diagnosed T2DM and having related diagnostic test results, a Modified Barthel Index score less than 100 for those who need care and 60 years old or older, and providing informed consent to participate. The exclusion criteria were as follows: people with cognitive or psychiatric disorders and other serious physical illnesses, as well as those taking part in other studies that could affect the findings of this one. At the same time, the inclusion criteria for the family caregivers were as follows: being the participant’s primary family caregiver and older than 18 years old, giving family members care without payment, providing the participant with care for 6 months or longer, and providing informed consent to participate. Before testing, the respondent is fully told of the survey’s relevance and aim, and their informed consent is gained by having them sign an informed consent form. The Jinzhou Medical University Ethics Committee had agreed to the study’s plan (Ethics Approval Number: JZMULL2022079).

### Data collection

The research team consisted of three graduate students, all of whom had passed uniform training. The researchers distributed questionnaires on site. The patients and family caregivers who met the inclusion criteria signed informed consent forms after explaining the purpose of the survey, requirements and filling methods of the questionnaire. The questionnaires were filled in anonymously, returned on the spot after completion, and answers were given to the respondents who did not understand the questionnaire. Patients and their family caregivers were timely informed of any omissions.

### Measures

#### Demographic characteristic

This section consists of two parts. The first asked demographic questions about the T2DM patients included gender, age, education, marital status, comorbidities, complications, etc. The second asked demographic questions about family caregivers, such as relationship to person with T2DM, duration of care, etc.

#### Modified barthel index

The Modified Barthel Index (MBI) is a modification of the Barthel Index (BI) with a higher sensitivity than the BI, and it is a significant assessment of activities of daily living (ADL) [22]. Therefore, this study used the MBI to assess diabetes patients and determine whether they require caregivers. As with the BI, the highest score of the MBI is 100, with higher scores indicating increased ADL. Severe dependence (≤ 40), moderate dependence (41–60), light dependence (61–99), and no dependence (100) are the classifications based on the overall score [23].

#### Quality of life

This study was evaluated the quality of life of T2DM patients with the Diabetes Specific Quality of Life Scale (DSQLS) [24], which contains four dimensions, physiological function, psychological, social and therapeutic dimension, with a total of 27 items. The higher the score, the lower the quality of life and the extent to which the disease had afflicted the patients. Among them, the total score of this scale is divided into three grades: general (total score ≥ 80), medium (total score 40–80), and good (total score ≤ 40). In the present study, Cronbach’s α of internal consistency of the scale was 0.737.

#### Caregiver competence

The Family Caregiver Task Inventory (FCTI) was used to measure caregiver competence among the participants in the present study. Compiled by Clark and Rakowski [25], later translated and revised by Lee [26], it consists of 25 items divided into five subscales. The scores of these 25 items range from 0 to 2 and the sum of each item represents the caregiver’s caring ability, with higher total scores representing greater difficulty level of caregiver tasks and lower care ability. In this study, the overall Cronbach’s α of the scale was 0.776.

#### Sense of coherence

The sense of coherence was measured with the SOC-13 questionnaire adapted from a longer 29-item scale [27]. It includes three dimensions: meaningfulness, comprehensibility, and manageability. The answer to the items is based on a seven-point Likert-type scale ranging from 1 to 7. Five variables are negatively worded and are therefore reverse-coded when summing the items to reach an item total. The overall score ranges from 13 to 91, with higher scores indicating that the individual possesses sense of coherence to a better degree. The overall Cronbach’s α of this scale was 0.750.

### Statistical analysis

All analyses were conducted using SPSS 25. The study population was described using mean ± standard deviation (Mean ± SD) and n (%). The relationships between sociodemographic factors and QoL scores of T2DM patients were expressed by T-test or one-way ANOVA analyses. Multivariate linear regression analysis was used to identify the independent factors associated with the QoL of patients. The Enter method is used in multiple linear regression. Pearson Correlation was used to measure the relationship between SOC and caregiver competence with QoL. The alpha level for significance in all analyses was determined to be *P*<0.05.

## Results

### Baseline characteristics of the participants

Table 1 describes the demographics of T2DM. Among the 392 T2DM, 64.8% of them were in the age groups of 60–70 years. Over half of the respondents was the man (58.4%), and almost all (79.8%) of the patients reported being married. In addition, 83.2% of the patients lived in urban areas and the educational level of the participants was junior or senior high school (63.5%). There were 183 patients (46.7%) with a disease course of 10–20 years and 265 patients (67.6%) with other health problems. In this study, the majority of patients (58.7%) have 1–2 complications and 38.3% of the patients were treated with both oral medications and insulin, followed by insulin therapy (36.2%), and only lifestyle modification, such as dietary control (2.6%). Moreover, most of them had an ADL score was 61–99 points and mild dependence was required in everyday life.

**Table 1:** Characteristics of T2DM patients that related to QoL (*n* = 392)

Variables	Overall sample n (%)	Quality of Life scores		
		Mean ± SD	t or F	*P* value
Sex			-2.051	0.041
Male	229 (58.4)	60.49 ± 7.35		
Female	163 (41.6)	62.04 ± 7.34		
Age (years)			28.966	<0.001
60–70	254 (64.8)	59.55 ± 6.79		
71–80	97 (24.7)	62.35 ± 6.99		
> 80	41 (10.5)	68.07 ± 7.31		
Marital status			-5.495	<0.001
Married	313 (79.8)	60.14 ± 6.98		
Single/divorced/widowed	79 (20.2)	65.06 ± 7.61		
Living area			3.704	<0.001
Rural	66 (16.8)	64.15 ± 6.76		
City	326 (83.2)	60.52 ± 7.35		
Level of education			37.458	<0.001
Primary and below	100 (25.5)	66.19 ± 6.91		
Junior or senior high school	249 (63.5)	59.43 ± 6.69		
College and above	43 (11.0)	59.26 ± 6.88		
Duration of T2DM (years)			15.263	<0.001
< 10	147 (37.5)	58.73 ± 7.03		
10–20	183 (46.7)	62.08 ± 6.88		
> 20	62 (15.8)	64.06 ± 7.97		
Other diseases			-0.954	0.341
No	127 (32.4)	60.62 ± 7.47		
Yes	265 (67.6)	61.38 ± 7.33		
Complications			24.893	<0.001
0	142 (36.2)	57.92 ± 6.67		
1–2	230 (58.7)	62.33 ± 6.70		
3–4	18 (4.6)	69.94 ± 7.74		
≥ 5	2 (0.5)	72.00 ± 15.56		
Type of treatment			1.466	0.223
Dietary control	10 (2.6)	59.60 ± 7.14		
Oral	90 (23.0)	60.12 ± 7.15		
Insulin	142 (36.2)	60.59 ± 6.92		
Oral-insulin	150 (38.3)	62.02 ± 7.88		
Activities in daily living			14.024	<0.001
Mild dependence (61–99)	380 (96.9)	60.80 ± 7.17		
Moderate dependence (41–60)	10 (2.6)	70.70 ± 5.74		
Heavy dependence (≤ 40)	2 (0.5)	76.50 ± 6.36		

The personal characteristics of family caregivers are presented in Table 2. The results showed that 61.2% of family caregivers were women and most of them were mostly aged 61–70 years. In this study, 98% of caregivers were married and 74.7% of the caregivers were spouses. Most of them had at least a junior or senior high school level of education (66.8%) and a monthly family income of around 3,000 RMB. Regarding the care information, 42.3% of caregivers with a length of care of 3–5 years, and more than half of caregivers spend an average of 4–8 h a day.

**Table 2:** Characteristics of family caregivers that related to QoL (*n* = 392)

Variables	Overall sample n (%)	Quality of Life scores		
		Mean ± SD	t or F	*P* value
Sex			3.530	<0.001
Male	152 (38.8)	62.76 ± 7.74		
Female	240 (61.2)	60.10 ± 6.95		
Age (years)			1.484	0.228
≤ 60	134 (34.2)	62.01 ± 7.50		
61–70	200 (51.0)	60.60 ± 7.30		
> 70	58 (14.8)	60.97 ± 7.28		
Marital status			0.004	0.997
Married	384 (98.0)	61.14 ± 7.42		
Single/divorced/widowed	8 (2.0)	61.13 ± 4.73		
Level of education			1.583	0.207
Primary and below	76 (19.4)	62.43 ± 7.16		
Junior or senior high school	262 (66.8)	60.73 ± 7.41		
College and above	54 (13.8)	61.26 ± 7.40		
Monthly family income (RMB)			0.148	0.863
< 3000	184 (46.9)	61.26 ± 7.46		
3000–5000	193 (49.2)	61.09 ± 7.27		
> 5000	15 (3.8)	60.20 ± 8.02		
Caregiver relationship to patient			20.475	<0.001
Spouse	293 (74.7)	59.85 ± 6.82		
Son or daughter	88 (22.4)	65.33 ± 7.54		
Relatives	11 (2.8)	61.14 ± 8.37		
Years of caring experience (years)			5.326	0.005
< 3	87 (22.2)	58.93 ± 6.67		
3–5	166 (42.3)	61.51 ± 7.20		
> 5	139 (35.5)	62.06 ± 7.76		
Time spent caring (h/day)			45.867	<0.001
< 4	153 (39.0)	57.50 ± 6.14		
4–8	219 (55.9)	62.93 ± 6.85		
> 8	20 (5.1)	69.30 ± 8.03		

### Factors associated with quality of life

Table 1 shows the association between the QoL of T2DM patients and the general demographic characteristics of study participants, including gender (t = -2.051, *P* = 0.041), age (t = 28.966, *P*<0.001), marital status (t=-5.495, *P*<0.001), living area (t = 3.704, *P*<0.001), level of education (t = 37.458, *P*<0.001), duration of T2DM (t = 15.263, *P*<0.001), complications (t = 24.893, *P*<0.001) and activities in daily living (t = 14.024, *P*<0.001). In addition, Table 2 also shows that the gender of the caregiver (t = 3.530, *P*<0.001), their relationship to the patient (t = 20.475, *P*<0.001), time spent caring (t = 5.326, *P* = 0.005) and average time spent caring (t = 45.867, *P*<0.001) are factors that are related to the QoL of the patient.

### Correlation between SOC, FCTI, and QoL in T2DM patients

The average score of SOC, FCTI, and QoL of T2DM patients and their correlation are shown in Table 3. Correlation analysis showed that there was a negative correlation between SOC and FCTI (*r*=-0.572, *P* < 0.01), SOC was negatively correlated with QoL (*r*=-0.344, *P* < 0.01), FCTI was positively correlated with QoL (*r* = 0.522, *P* < 0.01).

**Table 3:** Correlation between SOC, FCTI and QoL in T2DM patients (*n* = 392, Mean ± SD)

Variables	Mean ± SD	SOC r (*P*-value)	FCTI r (*P*-value)
SOC	55.17 ± 6.47	1	
FCTI	15.20 ± 4.78	-0.572**	1
QoL	61.14 ± 7.37	-0.344**	0.522**

SOC sense of coherence, FCTI family caregiver task inventory, QoL quality of life

**p < 0.01

### Predictors of QoL of T2DM patients

To investigate the crucial factors affecting the level of QoL of T2DM patients. The total score of QoL in patients was set as a dependent variable, the variables were subjected to a multifactorial analysis after excluding those that were highly correlated, based on the clinical context, and the results of the analysis showed that the age, ADL, duration of T2DM, SOC and caregiver competence had a significant effect on the QoL in T2DM patients, as shown in Table 4.

**Table 4:** Multiple linear regression analysis of QoL in T2DM patients

Variables	B	SE	β	t	*P*
(Constant)	43.786	4.590	-	9.540	0.000
Age	2.306	0.483	0.212	4.774	0.000
Activities in daily living	3.329	1.501	0.096	2.218	0.027
Duration of T2DM	1.622	0.434	0.154	3.736	0.000
SOC	-0.114	0.056	-0.100	-2.026	0.043
FCTI	0.629	0.078	0.408	8.017	0.000

SOC sense of coherence, FCTI family caregiver task inventory

## Discussion

This cross-sectional study examines the level of QoL of patients with T2DM and its relationship with general demographic characteristics of both the patient and the caregiver, the caregiver’s SOC, and caregiver competence. The data depicted that the total score of QoL among elderly patients with T2DM was 61.14 ± 7.37, which was similar to another study conducted in China that used the same measurement among patients with T2DM [28], and indicates that the patients with T2DM had a medium QoL in both studies, but higher than reported in an Iran and Ethiopia study [8, 29]. This difference might be explained by the improved financial status and the availability of primary medical insurance [30].

Variable results have been found in the literature addressing the QoL of patients with T2DM and its relationship to sociodemographic parameters. It is common knowledge that age has become a definitive factor in the QoL of patients [31, 32]. Research from other studies has confirmed our finding that younger patients had higher QoL scores [33]. Previous reports found that lower educational level, belonging to the female sex, long duration of illness, and complications were associated with poor QoL in patients with T2DM [34–36]. Similar to our finding, it may be that higher literacy levels in patients may possess better life skills, more capacity for managing their diseases, and superior informatics proficiency. However, with the prolongation of the course of the disease and complications arise, the patient’s insulin resistance is gradually weakened and the risk factors are increased, resulting in poor blood sugar control and overall decline in patients’ QoL. Our investigation, in contrast to other studies [37, 38], did not reach the conclusion that patients with comorbidities had lower QoL. This may be because the scale used in this study was more focused and less sensitive to the impact of other conditions on QoL [39] as well as individual reporting differences. Healthcare professionals must exercise extra caution when managing the comorbidities of T2DM, despite the fact that we did not find an effect, as other research has demonstrated that QoL deteriorates and lifespan dramatically declines as the number of comorbidities grows [40].

The factors that influence the QoL of people with T2DM are widely known, but this study argues that there is still a need to identify which of the variables attributable to carers may improve the QoL of patients. Therefore, our study included caregiver-related factors in the analysis as well. The study’s findings revealed that the patient’s QoL was impacted by the caregiver’s gender. As part of the custom, males typically hold positions of authority and control the family economy, while women are mostly in charge of managing the household’s daily operations, such as caring for children and elderly parents. Over time, they might develop their caregiving abilities and gain expertise [41]. Women are more inclined to adopt the position of caregiver and take on caring responsibilities when a family member is in danger of becoming unwell. In this study, 61.2% of the caregivers were female, and compared to male caregivers, they did a better job of enhancing patients’ quality of life. The results of this study suggest that patients show better QoL when the caregiver is their spouse. Our findings were in accordance with Potier [42]. A possible explanation is that some individuals can view providing care as a natural part of life and a responsibility to their families. These kinds of perceptions may be more prevalent in spouses than in sons or daughters [43]. In China, the current predicament facing the children of this generation is that of having both the old and children. They not only have to consider the health of the elderly and the raising of children, but also have to face their work, which will lead to a lack of energy under the multiple pressures, and the traditional Chinese filial piety will aggravate their anxiety, which will in turn increase the burden of caregiving and affect the quality of caregiving. In addition, patients typically have a lower QoL when their caregivers have been providing care for more than five years, on average for eight hours or more per day. The caregiver’s assumption of the caregiving task will have an impact on the original life, and with the prolongation of the caregiving time, the patient’s dependence on him or her increases, and the prolonged time spent facing the patient leads to stress and anxiety, which in turn affects the quality of care and leads to a lowering of the patient’s QoL [44].

This study confirmed that the SOC of the caregiver was negatively associated with patients’ QoL. But it can predict them positively. This is consistent with research from Taiwan that showed older patients with hip fractures were more likely to have a poorer QoL following surgery if their caregivers had lower psychological functioning [45]. This implies that when caregivers are in better physical and mental health, their actions give patients a sense of security and support, which influences their mood and boosts their self-assurance in beating the illness. All of this enhances the patients’ quality of life, particularly the psychological part of it. Furthermore, we also found that caregiver competence was positively correlated with patients’ QoL. In line with our findings, Huang et al. [46] studied hemodialysis patients and showed that patients’ QoL was associated with caregivers’ caregiver competence. This was also a consistent discovery in a study of elderly adults with disabilities [47]. High levels of caregiver competence among family caregivers indicate a certain amount of knowledge about illness susceptibility factors, fundamental skills, and preventing complications, all of which help to somewhat slow down the patient’s disease progression. In this study, family caregivers’ SOC was negatively correlated with, but positively predictive of, caregiver competence. A Dutch study reached a similar conclusion [48]: caregivers who experience low levels of SOC are more likely to believe that the care conditions they face are complex and challenging to comprehend and manage, and they may not be motivated to strive for higher levels of care.

Regression analysis showed that age, disease duration, ADL scores, SOC and FCTI were the major predictors of QoL in patients with T2DM. This finding supports research on another chronic disease [49], demonstrating that SOC has a positive predictive effect on the QoL of T2DM patients. SOC is expected to assist caregivers in making better use of resources. Those who care for others with an optimistic outlook on caring may be better able to handle a difficult situation. Antonovsky’s theory encourages people to reflect on stressful situations so that they can understand stress, think about how to make coping with them and identify the best ways to deal with them that make sense [14]. Therefore, one plausible explanation is that caregivers with a strong sense of coherence are better able to manage difficulties and chronic stress in the caregiving process, making the best use of the social resources available to them and enhancing caregiving capacity and quality of care [50]. A survey in Japan concluded that the sense of care burden was lower when the mental health status was high [51]. This means that high levels of psychological consistency in caregiving tend to be accompanied by less caregiving burden and higher levels of caregiving capacity. In this study, the QoL in T2DM was significantly predicted by caregiver competence as well. Nobahar et al. identified that the therapeutic effect and QoL of hemodialysis patients may be directly impacted by the caregivers’ ability to provide care [52]. Additionally, higher skill levels imply that carers can combine their own lives with caring responsibilities. This means that in the future, in addition to focusing on the mental health of family caregivers, we should also work to enhance the caregivers’ capacity to provide for their patients by coordinating resources for healthcare and promoting health in order to enhance the patients’ QoL.

T2DM is an incurable disease, so improving the QoL of patients is particularly crucial [53]. Elderly patients have an increased dependence on their caregivers and studies have confirmed that caregivers often have a direct or indirect impact on the QoL of patients [51, 54]. The above findings suggest that clinical interventions should give some attention to carers to improve patients’ quality of life in many ways.

The study’s limitations are convenience sampling and the cross-sectional nature of the study, both of which could not identify the causal link. Another potential drawback associated with the questionnaires’ self-reporting nature is recall bias. Finally, this study’s findings were derived from T2DM patients and their caregivers using healthcare resources from two hospitals in Jinzhou. The findings may, therefore, be transferable to different similar settings or conditions but are not generalizable to the population in a positivist sense.

## Conclusions

This is the first study that assessed the relationship between T2DM patients’ QoL and SOC and caregiver competence of caregiver. The results demonstrate a statistically significant relationship between T2DM patients’ enhanced QoL and their caregivers’ high SOC and caregiver competence. High SOC of caregivers typically has less difficulty providing care, are more competent to provide care, and have better patient outcomes. These new relationships could inform the development of clinical interventions to target the group of family caregivers to improve the QoL of patients.

### Electronic supplementary material

Below is the link to the electronic supplementary material.

Supplementary Material 1

## Data Availability

The datasets used and/or analyzed during the current study are available from the corresponding author upon reasonable request.

## Abbreviations

T2DM: Type 2 diabetes mellitus
SOC: Sense of coherence
QoL: Quality of life
FCTI: Family caregiver task inventory
ADL: Activities of daily living
