# Supplementary material for: Factors associated with quality of life among elderly patients with type 2 diabetes mellitus: the role of family caregivers
Source: BMC Public Health. 2024 Feb 21;24:539. doi: 10.1186/s12889-024-17917-z (PMC10880260; doi:10.1186/s12889-024-17917-z)
Supplement: Supplementary file 1 — Supplementary Material 1 [file 12889_2024_17917_MOESM1_ESM.docx]

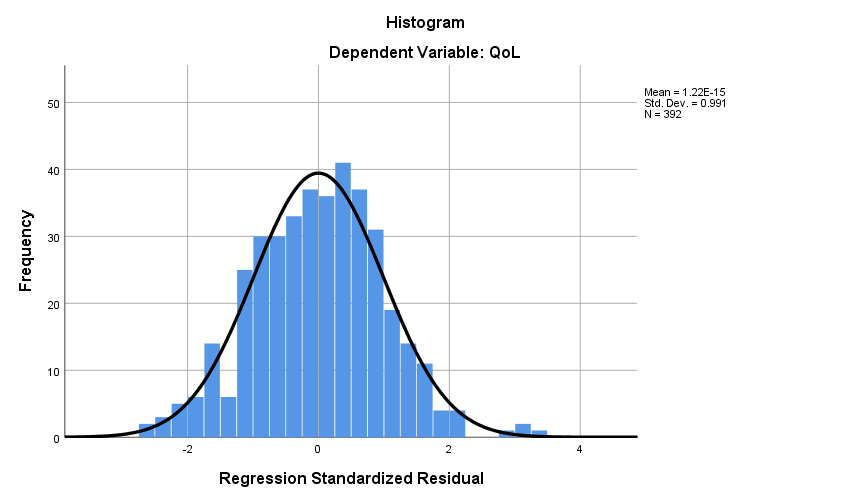


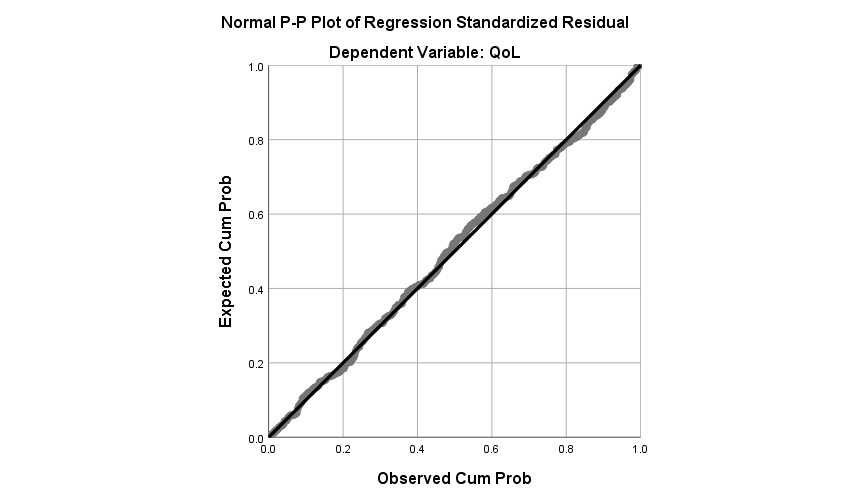


| **Residuals Statistics^a^** | | | | | |
| --- | --- | --- | --- | --- | --- |
|  | Minimum | Maximum | Mean | Std. Deviation | N |
| Predicted Value | 53.30 | 77.05 | 61.14 | 4.580 | 392 |
| Std. Predicted Value | -1.710 | 3.475 | .000 | 1.000 | 392 |
| Standard Error of Predicted Value | .467 | 2.839 | .769 | .320 | 392 |
| Adjusted Predicted Value | 53.32 | 76.14 | 61.13 | 4.584 | 392 |
| Residual | -15.833 | 19.444 | .000 | 5.776 | 392 |
| Std. Residual | -2.717 | 3.336 | .000 | .991 | 392 |
| Stud. Residual | -2.731 | 3.375 | .000 | 1.001 | 392 |
| Deleted Residual | -16.004 | 19.904 | .002 | 5.891 | 392 |
| Stud. Deleted Residual | -2.755 | 3.422 | .000 | 1.004 | 392 |
| Mahal. Distance | 1.513 | 91.786 | 6.982 | 9.774 | 392 |
| Cook's Distance | .000 | .056 | .003 | .005 | 392 |
| Centered Leverage Value | .004 | .235 | .018 | .025 | 392 |
| a. Dependent Variable: QoL | | | | | |

When the absolute value of the std. residual, stud. residual is greater than 3, the observation is considered an outlier. According to the table, then there are no outliers in the study.

| **Coefficients^a^** | | | | | | | | | | | |
| --- | --- | --- | --- | --- | --- | --- | --- | --- | --- | --- | --- |
| Model | | Unstandardized Coefficients | | Standardized Coefficients | t | Sig. | Correlations | | | Collinearity Statistics | |
|  |  | B | Std. Error | Beta |  |  | Zero-order | Partial | Part | Tolerance | VIF |
| 1 | (Constant) | 43.786 | 4.590 |  | 9.540 | .000 |  |  |  |  |  |
|  | age | 2.306 | .483 | .212 | 4.774 | .000 | .352 | .237 | .191 | .812 | 1.231 |
|  | Duration of T2DM | 1.622 | .434 | .154 | 3.736 | .000 | .266 | .187 | .149 | .946 | 1.057 |
|  | Activities in daily living | 3.329 | 1.501 | .096 | 2.218 | .027 | .258 | .112 | .089 | .862 | 1.160 |
|  | Marital status | 3.137 | 2.182 | .060 | 1.438 | .151 | .000 | .073 | .057 | .910 | 1.098 |
|  | Monthly family income | .757 | .558 | .058 | 1.355 | .176 | -.022 | .069 | .054 | .864 | 1.157 |
|  | SOC | -.114 | .056 | -.100 | -2.026 | .043 | -.344 | -.103 | -.081 | .655 | 1.527 |
|  | FCTI | .629 | .078 | .408 | 8.017 | .000 | .522 | .379 | .321 | .619 | 1.617 |
| a. Dependent Variable: QoL | | | | | | | | | | | |

| **ANOVA^a^** | | | | | | |
| --- | --- | --- | --- | --- | --- | --- |
| Model | | Sum of Squares | df | Mean Square | F | Sig. |
| 1 | Regression | 8203.453 | 7 | 1171.922 | 34.499 | .000^b^ |
|  | Residual | 13044.381 | 384 | 33.970 |  |  |
|  | Total | 21247.834 | 391 |  |  |  |
| a. Dependent Variable: QoL | | | | | | |
| b. Predictors: (Constant), FCTI, Marital status, duration of T2DM, activities in daily life, monthly family income, age, SOC | | | | | | |

| **Model Summary^b^** | | | | | | | | | |
| --- | --- | --- | --- | --- | --- | --- | --- | --- | --- |
| Model | R | R Square | Adjusted R Square | Std. Error of the Estimate | Change Statistics | | | | |
|  |  |  |  |  | R Square Change | F Change | df1 | df2 | Sig. F Change |
| 1 | .621^a^ | .386 | .375 | 5.828 | .386 | 34.499 | 7 | 384 | .000 |
| a. Predictors: (Constant), FCTI, Marital status, duration of T2DM, activities in daily life, monthly family income, age, SOC | | | | | | | | | |
| b. Dependent Variable: QoL | | | | | | | | | |

| **Correlations** | | | | | | | | | |
| --- | --- | --- | --- | --- | --- | --- | --- | --- | --- |
|  | | QoL | age | Duration of T2DM | Activities in daily life | Marital status | Monthly family income | SOC | FCTI |
| Pearson Correlation | QoL | 1.000 | .352 | .266 | .258 | .000 | -.022 | -.344 | .522 |
|  | age | .352 | 1.000 | .199 | .350 | -.097 | .041 | -.021 | .190 |
|  | Duration of T2DM | .266 | .199 | 1.000 | .087 | -.059 | .009 | -.099 | .135 |
|  | Activities in daily life | .258 | .350 | .087 | 1.000 | -.024 | .065 | -.092 | .155 |
|  | Marital status | .000 | -.097 | -.059 | -.024 | 1.000 | .269 | .108 | -.082 |
|  | Monthly family income | -.022 | .041 | .009 | .065 | .269 | 1.000 | .196 | -.228 |
|  | SOC | -.344 | -.021 | -.099 | -.092 | .108 | .196 | 1.000 | -.572 |
|  | FCTI | .522 | .190 | .135 | .155 | -.082 | -.228 | -.572 | 1.000 |
| Sig. (1-tailed) | QoL | . | .000 | .000 | .000 | .498 | .331 | .000 | .000 |
|  | age | .000 | . | .000 | .000 | .027 | .209 | .341 | .000 |
|  | Duration of T2DM | .000 | .000 | . | .042 | .123 | .432 | .025 | .004 |
|  | Activities in daily life | .000 | .000 | .042 | . | .315 | .101 | .034 | .001 |
|  | Marital status | .498 | .027 | .123 | .315 | . | .000 | .016 | .053 |
|  | Monthly family income | .331 | .209 | .432 | .101 | .000 | . | .000 | .000 |
|  | SOC | .000 | .341 | .025 | .034 | .016 | .000 | . | .000 |
|  | FCTI | .000 | .000 | .004 | .001 | .053 | .000 | .000 | . |
| N | QoL | 392 | 392 | 392 | 392 | 392 | 392 | 392 | 392 |
|  | age | 392 | 392 | 392 | 392 | 392 | 392 | 392 | 392 |
|  | Duration of T2DM | 392 | 392 | 392 | 392 | 392 | 392 | 392 | 392 |
|  | Activities in daily life | 392 | 392 | 392 | 392 | 392 | 392 | 392 | 392 |
|  | Marital status | 392 | 392 | 392 | 392 | 392 | 392 | 392 | 392 |
|  | Monthly family income | 392 | 392 | 392 | 392 | 392 | 392 | 392 | 392 |
|  | SOC | 392 | 392 | 392 | 392 | 392 | 392 | 392 | 392 |
|  | FCTI | 392 | 392 | 392 | 392 | 392 | 392 | 392 | 392 |
